# The γ _c_ family of cytokines: fine-tuning signals from IL-2 and IL-21 in the regulation of the immune response

**DOI:** 10.12688/f1000research.12202.1

**Published:** 2017-10-23

**Authors:** Rosanne Spolski, Daniel Gromer, Warren J. Leonard

**Affiliations:** 1 Laboratory of Molecular Immunology and the Immunology Center, National Heart, Lung, and Blood Institute, National Institutes of Health, Bethesda, Maryland, USA

**Keywords:** gamma chain cytokines, interleukins, IL-2, IL-21, XSCID

## Abstract

Interleukin (IL)-2, IL-4, IL-7, IL-9, IL-15, and IL-21 form a family of cytokines based on the sharing of a receptor component, the common cytokine receptor γ chain, γ _c_, which is encoded by the gene mutated in humans with X-linked severe combined immunodeficiency (XSCID). Together, these cytokines play critical roles in lymphoid development, differentiation, growth, and survival as well as mediating effector function. Here, we provide an overview of the main actions of members of this cytokine family but then primarily focus on IL-2 and IL-21, discussing their dynamic interplay and contributions to a fine-tuned immune response. Moreover, we discuss the therapeutic utility of modulating their actions, particularly for autoimmunity and cancer.

## Introduction: the γ _c_ system and its association with severe combined immunodeficiency

**Figure 1.**
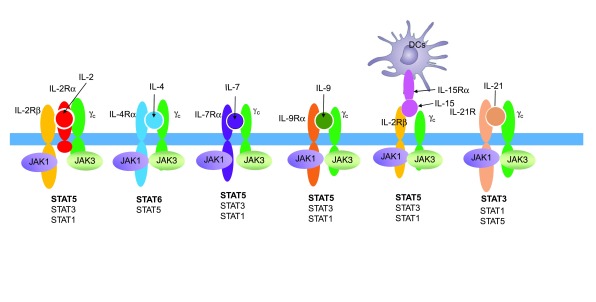
γ _c_ family cytokines and their receptors. Shown is the receptor for each γ _c_ family cytokine as well as the interacting Janus kinase 1 (JAK1) and JAK3 kinases and the signal transducer and activator of transcription (STAT) proteins activated by each cytokine. DC, dendritic cell; IL, interleukin.

Interleukin-2 (IL-2), IL-4, IL-7, IL-9, IL-15, and IL-21 form a family of four α-helical bundle type I cytokines that share the common cytokine receptor γ chain, γ _c_, as a key receptor component ( Figure 1) ^1^. γ _c_ is mutated in humans with X-linked severe combined immunodeficiency (XSCID), a disease in which T and natural killer (NK) cells are greatly diminished and B cells are non-functional ^2^. Finding the basis for XSCID immediately allowed more precise prenatal and postnatal diagnosis and carrier female identification as well as paving the way to gene therapy for this disease ^3, 4^. In addition, the implications of this finding extended far beyond the management of a single disease and had major basic scientific implications as well. Although γ _c_ was initially discovered as the IL-2 receptor γ chain (IL-2Rγ) and identifying the genetic basis for XSCID resulted from studies of the IL-2R, the fact that the phenotype in XSCID is more severe than in IL-2 deficiency led to the prediction and then discovery that IL-2Rγ was in fact a shared receptor component ^5– 8^, and the term γ _c_ was proposed ^5^. Interestingly, the major phenotypic abnormalities do not result from defective IL-2 signaling; instead, defective signaling by IL-7 and IL-15 explain the profound decrease in T and NK cells, respectively, and defective IL-21 signaling substantially explains the non-functional B cells in this disease ^9^. Thus, XSCID was established to be a disease of defective cytokine signaling ^10^.

Each γ _c_ family cytokine activates the Janus family tyrosine kinases (JAK)1 and JAK3, which then trigger signaling cascades. JAK1 associates with the more distinctive type I cytokine receptor for each cytokine and JAK3 associates with γ _c_^11^. Because JAK3 is “downstream” of γ _c_, it was hypothesized ^12^ and then established that *JAK3*-deficient SCID indeed occurs, causing a T ^–^B ^+^NK ^–^ form of SCID that phenocopies XSCID ^13, 14^. Moreover, individuals with *IL7R*-deficient SCID were identified based on the prediction that such patients would have defective T cell development but that NK cells would develop normally given intact IL-15 signaling ^15^. Although IL-7 signaling is believed to be responsible for the lack of T cell development, to date human *IL7*-deficient SCID has still not been identified. Presumably, the frequency of inactivating mutations of *IL7* is very low, the condition is unexpectedly lethal, or the phenotype is less severe than anticipated so that individuals do not come to medical attention. A less severe phenotype for *IL7* than *IL7R* deficiency is conceivable given that signaling by thymic stromal lymphopoietin (TSLP) would also be affected in the latter but not the former, and indeed there is even more defective T cell development in mice lacking the expression of both γ _c_ and the TSLP receptor ( *Il2rg/Crlf2* double knockout mice) than in *Il2rg* single knockout mice ^16^. However, XSCID and *JAK3*-deficient patients have intact TSLP signaling but nevertheless have essentially absent T cell development, minimizing this as an explanation.

## STAT activation and major biological effects of γ _c_ family cytokines

γ _c_ family cytokines collectively have broad actions ( Table 1) and activate three major signaling pathways, including the MAP kinase, PI 3-kinase, and JAK–STAT (signal transducer and activator of transcription) pathways. Of the seven STAT proteins, IL-2 mainly activates STAT5A and STAT5B, but it also signals through STAT1 and STAT3 to some degree ^17^ (see Figure 1 for STATs activated by each γ _c_ family cytokine). It is a T cell growth factor that additionally augments the cytolytic activity of CD8 ^+^ T cells and NK cells and is essential for regulatory T (Treg) cell development ^18^. It also promotes the differentiation of T helper type 1 (Th1) ^19^, Th2 ^20, 21^, and Th9 cells ^22^ while inhibiting Th17 cell differentiation ^19, 23^. Importantly, from a clinical perspective, IL-2 exhibits anti-cancer activity and is approved by the FDA for the treatment of melanoma and renal cell carcinoma ^24^.

**Table 1.** Actions of γ _c_ family cytokines.

IL-2	• Promotes Th1, Th2, and Th9 differentiation and antagonizes Th17 and Tfh differentiation • Induces T cell and NK cell proliferation • Enhances Treg cell differentiation and function • Anti-cancer role for immunotherapy
IL-4	• Promotes B cell differentiation and Ig isotype switching • Promotes Th2 and Th9 differentiation • Proliferative effects on tissue-resident macrophages • Protection from helminth infection
IL-7	• Required for T cell development and homeostasis • Promotes memory CD8 ^+^ T cell development • Essential for B cell development in mice but dispensable for B cell development in humans
IL-9	• Promotes mast cell proliferation • Augments mucus production by goblet cells • Anti-tumor activity
IL-15	• Essential for NK development, expansion, and survival • Promotes memory CD8 ^+^ T cell development • Anti-cancer role for immunotherapy via actions on CD8 ^+^ T cells and NK cells
IL-21	• Promotes B cell differentiation to plasma cells and augmenting Ig production • Has anti-cancer activity mediated in part via actions on CD8 ^+^ T cells and NK cells • Promotes Tfh differentiation and germinal center formation • Promotes Th17 differentiation • Inhibits Th9 differentiation • Promotes autoimmune disease (type I diabetes, SLE, EAE, and colitis)

EAE, experimental autoimmune encephalomyelitis; Ig, immunoglobulin; IL, interleukin; NK, natural killer; SLE, systemic lupus erythematosus; Tfh, T follicular helper; Th, T helper; Treg, T regulatory.

IL-4 mainly activates STAT6 and plays major roles in allergic responses, including asthma and in protection against helminth infections ^25– 28^. IL-4 signals via two types of receptor. Type I IL-4 receptors (IL-4Rs) comprise IL-4R plus γ _c_^6^ and are expressed mainly on lymphoid cells. In contrast, type II IL-4Rs comprise IL-4R plus IL-13Rα1 (but not γ _c_) and are mainly expressed on non-lymphoid cells; these IL-4Rs also represent the functional IL-13 receptor ^28, 29^.

IL-7 is a stromal factor which, like IL-2, predominantly activates STAT5A and STAT5B ^17, 30^. It drives T cell development as well as normal CD8 ^+^ T cell homeostasis, particularly of memory CD8 ^+^ T cells. IL-7 is a potent survival factor for T cells, strongly inducing the expression of BCL2 ^31– 34^. Unlike other γ _c_ family cytokines, IL-7 is produced within the stroma and is more constitutively expressed.

IL-9 also activates STAT5A and STAT5B ^17, 30^. This cytokine can promote the expansion of mast cells. Interestingly, a single nucleotide polymorphism (SNP) in the *IL9R* gene associates with a haplotype that is protective against wheezing in boys but not in girls ^35^; this sex-related difference makes sense given that *IL9R* is located on the X chromosome. IL-9 also promotes anti-tumor immunity ^36, 37^.

Like IL-2, IL-15 primarily activates STAT5A and STAT5B ^17^ and shares IL-2Rβ as well as γ _c_ as receptor components. Like IL-2, IL-15 also has a specific α chain, IL-15Rα, so that these two cytokines each have three receptor components. However, whereas IL-2 signals mainly *in cis* by interacting with high-affinity IL-2 receptors that contain IL-2Rα, IL-2Rβ, and γ _c_ or intermediate-affinity receptors comprising IL-2Rβ and γ _c_, IL-15 primarily signals via trans-presentation of IL-15Rα-bound IL-15 to cells expressing IL-2Rβ and γ _c_^38^. IL-15 is critical for the development and expansion of NK cells as well as for memory CD8 ^+^ T cell homeostasis ^39, 40^.

IL-21 is the most recently identified γ _c_ family cytokine. IL-21 has pleiotropic actions, driving the terminal differentiation of B cells to plasma cells ^41, 42^ and cooperating with IL-7 and IL-15 to expand CD8 ^+^ T cells ^43^. Moreover, IL-21 serves a key role in promoting T follicular helper (Tfh) cell differentiation ^44^ and can augment Th17 differentiation *in vitro* ^45– 47^. Furthermore, IL-21 has anti-cancer activity in animal models and is being evaluated in human clinical trials. In addition, a broad range of animal models indicate that IL-21 plays a key role in the development of autoimmune disease, including for type 1 diabetes, systemic lupus erythematosus, and experimental autoimmune uveitis ^48^. Collectively, γ _c_ family cytokines therefore play broad and important biological roles, many of which are associated with diseases and may represent targets for therapeutic application. Below, we will focus on IL-2 and IL-21, which exhibit both overlapping and opposing actions.

## IL-2 and IL-21

IL-2 and IL-21 are encoded by adjacent genes on human chromosome 4q27 and mouse chromosome 3, and their structural homology suggests that these two genes may have arisen from a gene duplication event during evolution. Despite their proximity, the *IL2* and *IL21* genes are differentially regulated, and analysis of the chromatin region between these genes has revealed the presence of insulator regions that ensure their independent regulation ^49, 50^. IL-2 and IL-21 exert distinctive actions on immune cell populations, sometimes with opposite outcomes, which results, at least in part, from their differential activation of STAT proteins. Although both cytokines can activate STAT1, STAT3, STAT5A, and STAT5B, IL-2 predominantly activates STAT5A and STAT5B, whereas IL-21 mostly signals via STAT3. Consistent with these signaling differences, analysis of the effects of IL-2 and IL-21 on the anti-tumor activity of CD8 ^+^ T cells revealed that these cytokines induced distinctive transcriptional profiles, with associated differences in disease outcome ^51^. Whereas IL-2 enhanced CD8 ^+^ T cell proliferation and effector function, IL-21-treated cells exhibited a central memory phenotype, with greater persistence of the cells and higher anti-tumor activity *in vivo*. IL-2 and IL-21 also have markedly different effects on the *in vivo* function of Tfh cells and the *in vitro* differentiation of Th9 cells, as we discuss below. As continued investigation yields more insights into the mechanistic underpinnings of these two cytokines, investigators may learn how to specifically enhance the “good” features and inhibit the “bad” features of each. Here, we review recent advances in our understanding of IL-2 and IL-21, how they regulate multiple lymphoid populations, and potential strategies for utilizing the strengths of each cytokine in the treatment of disease.

## IL-2 signaling

As noted above, each γ _c_ family cytokine can activate the JAK–STAT pathway, but these cytokines collectively also activate phosphoinositide (PI) 3-kinase and extracellular signal-regulated kinase (ERK)-dependent pathways as well ^18^. The relative kinetics and potency of activation of JAK–STAT, PI 3-kinase, and ERK pathways and the kinetics of their activation are critical for determining the specificity of signaling. Each of these three major signaling pathways involves kinases, and IL-2, like other cytokines and growth factors, influences the exchange of phosphate groups. Understanding the mechanisms that these intracellular systems use to initiate specific differentiation programs, often by increasing the expression of key transcriptional regulators, represents a major step towards creating future interventions to alter cell fate ^18^.

Using mass spectrometry, a phosphoproteomic signature was recently identified in pre-activated CD8 ^+^ T cells that were cultured with IL-12 to maintain viability and then stimulated with IL-2 ^52^. IL-2 was shown to induce the phosphorylation and dephosphorylation of a large number of proteins that carry out vital functions, including transcription, RNA stabilization, nuclear translocation, protein translation, cell trafficking, metabolism, and cell cycle, thus potentially identifying new targets for manipulating IL-2 signaling. Surprisingly, inhibition of JAK3 and JAK1 signaling with tofacitinib affected only 4% of the phosphoproteome, suggesting that many of these phosphorylation events were independent of JAK signaling ^52^. Interestingly, Treg cells have been shown to express high levels of phosphatase and tensin homolog (PTEN) ^53^, which inhibits IL-2-induced PI 3-kinase signaling but does not affect STAT5 activation. PTEN thus may be differentially important in the control of IL-2-mediated Treg cell versus effector T cell function. Consistent with this possibility, the absence of PTEN can reduce IL-2Rα and FoxP3 expression by Treg cells, leading to autoimmune disease ^53^.

Investigators have also studied IL-2 diffusion through cell “niches” as measured by STAT5 phosphorylation in target cells ^54^. By using IL-2/anti-IL-2 complexes to expand Treg cells *in vivo* and assessing STAT5 signaling in conventional T cells, it was shown that the dimensions of cytokine gradients changed rapidly, depending on the number of cells (e.g., Treg cells) that were consuming cytokine. The IL-2 gradients identified suggest that there may be functional heterogeneity in response to antigen and cytokine signals, depending on the position of responding cells in three-dimensional space within a given organ ^54^, with possible therapeutic implications for manipulating IL-2 concentrations *in vivo*.

## Manipulating IL-2 signals in immunotherapy

The anti-cancer activity of IL-2 has long been known, and high-dose IL-2 can be toxic and is associated with capillary leak syndrome; thus, efforts to lower the toxicity have focused in part on lowering IL-2 ^24^. IL-2 can also promote activation-induced cell death (AICD), an unwanted effect that can be diminished by lowering doses of IL-2. Previous studies showed that in addition to it inducing the proliferation of CD8 ^+^ T cells, IL-2 preferentially stimulates short-lived effector T cells that are detrimental to cancer immunotherapy ^51^. IL-15 is another important cytokine for immunotherapy that, unlike IL-2, does not bind to IL-2Rα and thus does not preferentially stimulate Treg cells. Unlike IL-2, IL-15 is not associated with capillary leak syndrome nor does it mediate AICD ^55, 56^. Despite these potential advantages for IL-15, IL-2–anti-IL-2 complexes were superior to IL-15–soluble IL-15Rα complexes at supporting the anti-tumor activity of transferred CD8 ^+^ T cells ^57^. One basis for this could be that IL-15 is quickly internalized and mediates only brief STAT5 signaling in a lymphoreplete host, whereas IL-2 remains in a surface “reservoir”, trapped by excess IL-2Rα and recycled after internalization, which results in sustained STAT5 signaling ^57^. Indeed, a membrane-tethered form of IL-15 on tumor-specific T cells demonstrated improved T cell survival and enhanced anti-tumor effects *in vivo* due to the preferential growth of T memory stem cells ^58^. Thus, both IL-2 and IL-15 show considerable potential that is worthy of additional investigation.

**Figure 2.**
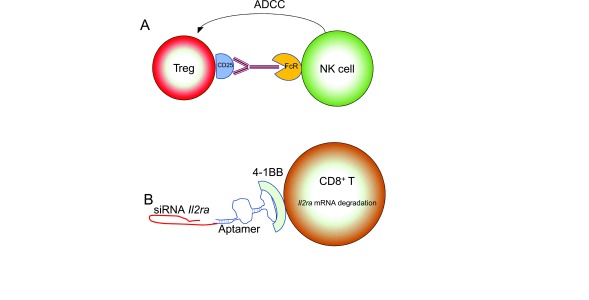
Schematic for mechanisms of manipulating interleukin (IL)-2 signals. ( **A**) CD25-specific antibody with an activating Fc region binds to CD25 on regulatory T (Treg) cells and activates natural killer (NK)-mediated antibody-dependent cell-mediated cytotoxicity (ADCC) via stimulation of the FcR. ( **B**) Silencing RNAs to *Il2ra* are conjugated to an aptamer that allows specific binding to 4-1BB on CD8 ^+^ T cells, allowing delivery specifically to activated cells. siRNA, small interfering RNA.

Another strategy for improving immunotherapeutic outcomes in malignancy involves disabling or eliminating tumor-associated Treg cells. Effector T cells within tumors have much lower expression of IL-2Rα (CD25) than do Treg cells, suggesting that targeting CD25 might be a therapeutically useful approach for preferentially depleting Treg cells ^59^. Although earlier attempts to deplete intratumoral Treg cells with antibodies to CD25 were not successful ^60, 61^, a new CD25-directed antibody with enhanced binding to an activating Fc region allowed Treg cell-specific antibody-dependent cell-mediated cytotoxicity (ADCC) ( Figure 2A), and when combined with a programmed death-1 (PD-1) blockade in mice, this treatment skewed the tumor-infiltrating lymphocyte (TIL) landscape towards activated, conventional T cells and improved rejection of established tumors ^59^.

Because IL-2 drives CD8 ^+^ T cells toward an “effector” phenotype, which confers poor anti-tumor performance in some models, reducing IL-2 signaling favors the production of memory CD8 ^+^ T cells ^62, 63^. A new strategy for reducing IL-2 signaling is to conjugate *Il2ra* siRNAs to a 4-1BB-binding oligonucleotide aptamer. Since 4-1BB is expressed on activated CD8 ^+^ T cells, this approach was designed to decrease IL-2 signaling specifically on these cells ( Figure 2B). ****Indeed, this aptamer increased “central memory” phenotype cells *in vitro* and enhanced tumor rejection in mice, demonstrating its potential efficacy ^64^.

Above, we discussed ways of modulating IL-2 signaling by efforts to diminish the expression of CD25 in conventional T cells, deplete CD25 ^high^ Treg cells, or to increase the availability of IL-2, either locally or systemically. The effects of complexes of IL-2 and IL-2-specific antibodies have also been studied. One monoclonal antibody binds with high affinity to human IL-2 at the CD25-binding epitope, thus preventing interaction with high-affinity receptors. Instead, the stabilized IL-2–anti-IL-2 complex preferentially acts on cells with a high level of IL-2Rβ-γ _c_ intermediate-affinity receptors, such as NK and CD8 ^+^ T cells, inducing their proliferation and STAT phosphorylation, decreasing TIL markers of “exhaustion”, and improving anti-tumor responses ^65^. Other IL-2–anti-IL-2 complexes can selectively stimulate either Treg or effector T cells, and protein interaction modeling coupled with biological experiments allows the engineering of therapeutic antibodies directed at specific immune subpopulations ^66^.

Besides the anti-cancer actions of IL-2, augmenting *in vivo* T cell exposure to IL-2 might be beneficial in vaccine strategies or in efforts to treat autoimmune disease. In one approach, IL-2 plasmid was co-administered with human papillomavirus (HPV) vaccination of mice, which increased the proliferation and effector differentiation of HPV E7-specific CD8 ^+^ T cells and their production of interferon (IFN)γ. Importantly, this augmented the ratio of effector cells to Treg cells, with an enhanced anti-tumor response ^67^.

Another approach to enhance the activity of IL-2 was to develop an IL-2 superkine with augmented affinity for IL-2Rβ ^68^. Normally, IL-2 first binds IL-2Rα, resulting in a conformational change in IL-2 that allows it to then efficiently bind IL-2Rβ; the IL-2–IL-2Rβ complex then efficiently recruits γ _c_. The IL-2 superkine "locks in" the altered conformation that normally results after binding IL-2Rα so that it efficiently binds to IL-2Rβ even in the absence of IL-2Rα ^68^. This superkine had increased activity, even at a low concentration, with decreased capillary leak syndrome ^68^. Derivatives of the IL-2 superkine have also been generated in order to fine-tune IL-2 signaling (discussed below) ^69^.

Using IL-2 to preferentially expand Treg cells constitutes an area of intense focus related to autoimmune disease. For example, providing low doses of IL-2 to patients with systemic lupus erythematosus (SLE) can increase the percentage of circulating Treg cells and may prove clinically beneficial ^70^. An initial study in which patients with SLE were given low-dose IL-2 reported an increase in the number of Treg cells, which was associated with an apparent decrease in a clinical SLE disease index ^71^, suggesting that such an approach may warrant evaluation in future clinical trials. A similar IL-2 regimen in mice resulted in augmented PD-1 expression in an “activated-memory” Treg cell subset, and PD-1 blockade resulted in apoptosis of these cells. Low-dose IL-2 treatment of humans with graft-versus-host disease was originally found to lead to expansion of Treg cells and reduced symptoms in a group of patients ^72^. Moreover, humans with graft-versus-host disease receiving low-dose IL-2 treatment could be retrospectively divided into likely responders and non-responders based on PD-1 expression on their peripheral Treg cells ^73^.

## Effects of IL-21 on germinal center T cells

IL-21 is a key regulator of T follicular helper (Tfh) cell development in germinal centers and represents a major cytokine secreted by Tfh cells that critically regulates the differentiation of memory B cells and plasma cells ^44^. Tfh cells appear to be heterogeneous with regard to their cytokine profiles and their anatomical location ^74, 75^. For example, when IL-4–IL-21 double reporter mice were infected with *Nippostrongylus brasiliensis*, few Tfh cells produced both IL-4 and IL-21, and those producing either IL-4 or IL-21 were localized to different regions of the germinal center. Tfh cells expressing IL-4, IL-21, or both cytokines also had distinct transcriptional profiles ^76^. Interestingly, in the germinal center, IL-21-producing Tfh cells, which are localized at a region involved in immunoglobulin (Ig) hypermutation, can differentiate into IL-4-producing Tfh cells, which are localized in an area more involved in the differentiation of plasma cells. When these individual Tfh populations were transferred into mice and immunized, Tfh cells producing IL-21 or IL-21 plus IL-4 induced higher expression of B-cell lymphoma 6 protein (BCL6) in the germinal center, with the cells producing both cytokines inducing a greater increase in germinal center size and more plasma cells ^76^, consistent with the synergistic effects of IL-4 and IL-21, as was first shown for immunoglobulin (Ig) production ^9^.

Tfh cells do not appear to be restricted to classical secondary lymphoid organs, as a population of these cells has also been identified in Peyer’s patches of the intestine ^77^. These Tfh cells produce high levels of IL-21, which is essential for the production of IgG1 by germinal center B cells in the intestine. Treatment of mice with antibiotics led to a dramatic decrease in the number of Tfh cells within the Peyer’s patches, indicating that an intact gut microbiome is required for the maintenance of these cells. Not only are Tfh cells capable of differentiating or migrating outside of the spleen and lymph nodes but they can also can exhibit functional plasticity. For example, when animals were exposed to house dust mite allergen and IL-21-expressing Tfh cells from these mice were then transferred into other primed mice, they migrated to the lung where they lost expression of IL-21 and differentiated into effector Th2 cells that expressed both IL-4 and IL-13 ^78^.

Interestingly, high production of IL-21 has been detected in populations of Tfh and Tfh-like cells that are external to germinal centers, and these cells can also regulate B cell activation and Ig production. For example, high levels of peripheral Tfh cells that secrete high levels of IL-21 have been found in a subset of HIV-infected patients, and the presence of this population correlated with an effective response to influenza vaccine ^79^. In addition, a population known as T peripheral helper (Tph) cells, comprising 30% of synovial fluid CD4 ^+^ T cells in rheumatoid arthritis patients, expresses chemokine receptors (CCR2, CX3CR1, CCR5) that allow the cells to migrate to sites of inflammation ^80^. In contrast to Tfh cells localized in germinal centers, Tph cells are PD-1 ^hi^ but are not exhausted, and they express high levels of B lymphocyte-induced maturation protein 1 (BLIMP1) but low levels of BCL6. Moreover, Tph and Tfh cells have not been interconverted *in vitro*, suggesting that Tph cells develop *in vivo* to induce B cell responses in pathological situations, such as within inflamed synovium ^80^.

IL-21 can also influence another small population of cells in the germinal center, known as T follicular regulatory (Tfr) cells, which negatively regulate Tfh-directed germinal center responses ^81– 83^. These Tfr cells share some phenotypic markers (CXCR5 ^+^BCL6 ^+^ICOS ^+^PD1 ^+^) with Tfh cells, but they also express the transcription factor FoxP3. Tfr cells can interact directly with Tfh cells to suppress their production and secretion of IL-21 and IL-4, thereby decreasing B cell Ig production ^84^. Tfr cells can also interact directly with B cells in the germinal center, inhibiting several metabolic pathways and diminishing their effector function ^84^. IL-21 can overcome the effects of Tfr, both by inhibiting their proliferation and by upregulating glycolysis in B cells, making them resistant to suppression by Tfr. Although IL-4 plays an important role in germinal center B cell responses, unlike IL-21, it cannot overcome Tfr-mediated suppression ^84^.

## IL-21 versus IL-2 effects on regulating CD4 ^+^ T cell responses within the germinal center

**Figure 3.**
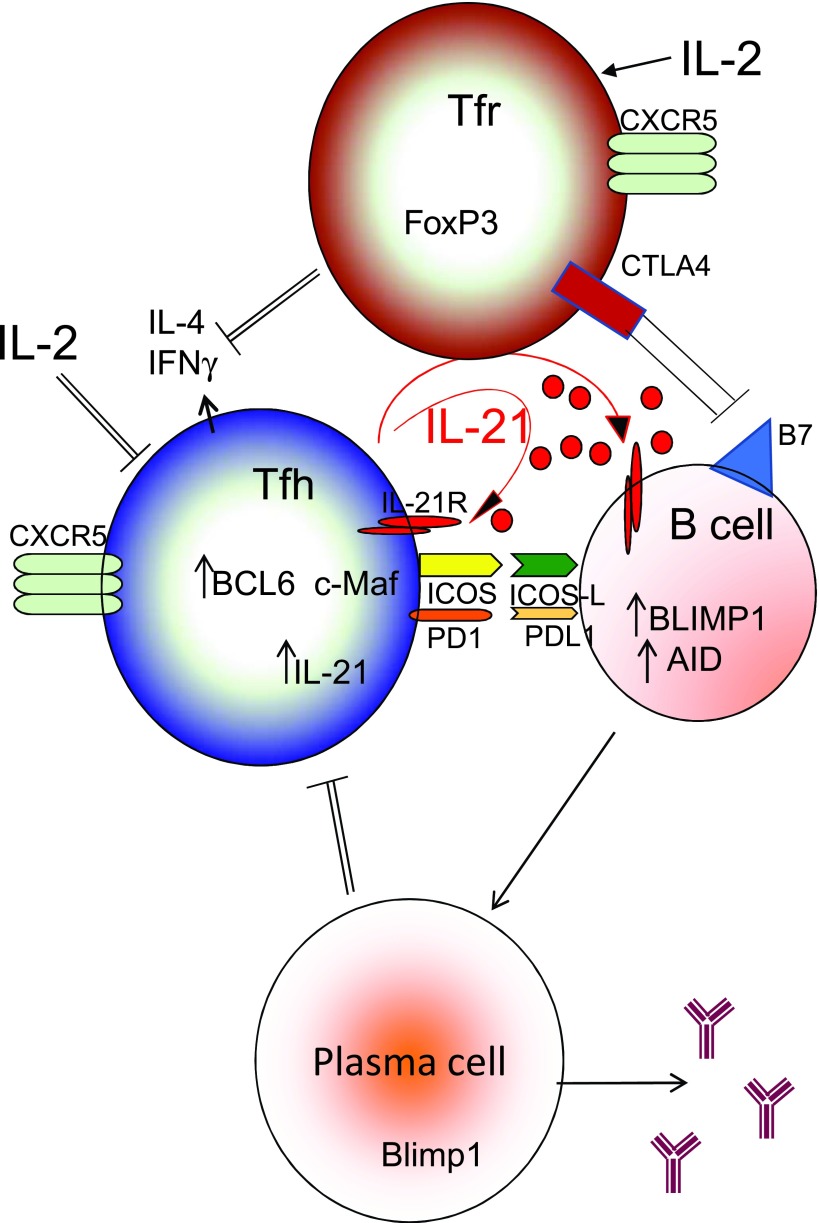
Roles of interleukin (IL)-2 and IL-21 in the regulation of germinal center development and function. IL-21 drives T follicular helper (Tfh) differentiation and function through the upregulation of B-cell lymphoma 6 protein (BCL6); moreover, it also induces the differentiation of B cells to immunoglobulin (Ig)-producing plasma cells through the upregulation of the B lymphocyte-induced maturation protein 1 (BLIMP1). IL-2 negatively regulates this process by inhibiting Tfh cell generation through the repression of BCL6 and also by inducing the function of T follicular regulatory (Tfr) cells that can directly interact with and inhibit B cell differentiation. AID, activation-induced deaminase; CTLA4, cytotoxic T lymphocyte-associated molecule-4; CXCR5, C-X-C chemokine receptor type 5; ICOS, inducible costimulator ligand; IFN, interferon; IL, interleukin; PD1, programmed death protein 1; PDL1, programmed death ligand 1.

As noted above, IL-2 and IL-21 are differentially regulated and have distinct effects on immune responses. Indeed, these cytokines may even have opposing effects on the formation of the cell populations within the germinal center that contribute to the generation of a humoral response to pathogens. Within the germinal center, Tfh and B cells interact with each other via IL-21 and ICOS:ICOS ligand interactions, leading to high expression of BCL6 by Tfh cells and the specification of the Tfh cell transcriptional profile ( Figure 3). Although IL-2 can expand effector T cell populations ^18^, interestingly, it can suppress the generation of Tfh cells within the germinal center in an influenza infection model ^85^. This inhibitory effect of IL-2 on Tfh cell generation was not due to the accumulation of Treg cells but rather was attributed to the ability of IL-2 to induce BLIMP1, which suppresses BCL6. Thus, whereas IL-21 induces BCL6 and promotes the accumulation of Tfh cells, IL-2 suppresses BCL6 and inhibits the germinal center response.

Interestingly, mounting evidence suggests that CD4 ^+^ T cells destined to become Tfh cells are guided to a niche where IL-2 cannot alter their course of differentiation. These cells exhibit a G-protein-coupled receptor, EBI2, that promotes migration to the “outer T zone”, and they co-localize with CD4 ^+^ dendritic cells that consume free IL-2 with soluble and transmembrane CD25 ^86^. This is consistent with the evolving model of antagonistic actions for IL-2 and IL-21 at the germinal center and that opposing effects of IL-2 and IL-21 are physiologically relevant.

Other studies have used an acute lymphocytic choriomeningitis virus (LCMV) infection model to dissect the dynamics of signaling and metabolism in Tfh and Th1 cells. As noted above, IL-2 inhibits Tfh differentiation but promotes Th1 differentiation. Activation of AKT, PI 3-kinase, and the mechanistic target of rapamycin (mTOR) by IL-2 was necessary for the generation of optimal levels of phospho-S6 and BLIMP-1, which by repressing the transcription of BCL6 can decrease Tfh cell differentiation. Additionally, the expression of T-Bet, a master regulator of Th1 differentiation, depends on AKT and Raptor (mTORc1) ^87^. Interestingly, Tfh cells in the LCMV infection model relied more on mitochondrial oxidation pathways and were less proliferative and less glycolytic than Th1 cells, although the significance of these observations remains unclear.

Although IL-2 signaling inhibits the germinal center response, as noted above, IL-2 induces the survival and function of Treg cells, which suppress both humoral and cellular immune responses ^88^. It was therefore surprising that when FoxP3 ^+^ Treg cells were depleted from mice prior to influenza infection, the Tfh cell response to virus infection was greatly reduced ^89^. Without Treg cells to bind and consume IL-2, more IL-2 was available to suppress Tfh cell generation and functional germinal center responses. Thus, under steady-state conditions, Treg cells with their high CD25 expression can compete for excess IL-2 in the follicle and promote the Tfh response. However, when IL-2 levels are sufficiently high, Tfh cell generation is suppressed.

Above, we showed an interplay between IL-21 and IL-2 related to Treg cells and Tfh cells, and indeed previous studies in mice had demonstrated that IL-21 inhibited Treg expansion after viral infection ^90^. While IL-2 enhances the survival of FoxP3 ^+^ Treg cells, IL-21 diminishes IL-2 production by conventional T cells and thereby lowers Treg cell numbers ^91^. As a result, one would predict that humans depleted of IL-21 would have increased Treg cells. In fact, *IL21R*-deficient patients have high numbers of both Treg cells and Tfr-like cells in their peripheral blood, suggesting that these populations are normally negatively regulated by IL-21 ^92^. Mechanistically, IL-21 induces BCL6 and lowers *IL2RA* gene expression, leading to a decrease in the ability of inhibitory Treg and Tfr cells to proliferate in response to IL-2, with a correspondingly more robust germinal center antibody response ^92^.

## Opposing actions of IL-2 and IL-21 in Th9 differentiation

IL-2 and IL-21 can drive alternative and sometimes opposing differentiation programs in a range of cell types, including the stimulatory and inhibitory germinal center populations noted above. Another example of opposing actions for these cytokines is in the differentiation of IL-9-producing Th9 cells. These cells, which were initially characterized as a population generated by stimulation with transforming growth factor (TGF)β and IL-4, have been shown to play a role in multiple inflammatory disease processes as well as in anti-tumor responses ^93^. IL-2 is known to promote the development of IL-9-producing cells ^94^, and details of the mechanisms involved have been elucidated ^22, 95^. IL-2-mediated activation of STAT5 is required for IL-9 production, with STAT5 binding at the *Il9* promoter. In contrast, IL-21 negatively regulates the initial production of IL-9, at least in part owing to the induction of transcription factor BCL6, which binds to the *Il9* promoter in close proximity to STAT5, suggesting that STAT5 and BCL6 compete for access to these promoter sites ^22^. Consistent with direct effects of IL-2-induced STAT5 signals on IL-9 transcription, mice deficient for *Itk*, a Tec family kinase activated via the T cell receptor, had defective production of interferon regulatory factor 4 (IRF4) and IL-9 production, and the expression of these factors could be rescued by IL-2 or constitutively activated STAT5 ^96^.

Unlike STAT5, STAT3 activation has been shown to negatively regulate both the initiation and the maintenance of IL-9 expression. Th9 cells subjected to multiple rounds of *in vitro* differentiation produced increasing amounts of IL-21 and IL-10, which then led to the extinction of IL-9 production ^97^. It remains to be determined whether this effect is relevant *in vivo*, but in this regard, IL-1β could induce the production of high levels of IL-21 by Th9 cells, but these cells nevertheless continued to produce IL-9 and acquired potent IL-21-mediated anti-tumor activity ^98^.

IL-2 was also reported to contribute to the pathogenic role of IL-9 in lung disease and inflammation in cystic fibrosis through a self-amplifying circuit involving IL-2 ^99^. In this circuit, lung epithelial damage resulted in the release of IL-33, which induced the expansion of innate lymphoid cells (ILCs) and their production of IL-9, which triggered mast cells to secrete IL-2. The mast cell-produced IL-2 then further expanded both CD25 ^+^ ILC2s as well as Th9 cells in the lung, promoting the ongoing inflammatory process.

## Fine-tuning of cytokine signals

Above we have discussed a range of physiological effects and potential therapeutic approaches using IL-2 and IL-21 and provided examples where they can oppose each other’s actions. These are illustrative, and many of the lessons learned in these studies can be extended to other systems. It is important to underscore that differential actions for single cytokines have been noted and fine actions of the cytokines can be attributed to the utilization of different STAT proteins—for example, where one cytokine exhibits the induction of different genes depending on which STAT is activated. For instance, IL-21 mainly acts via the activation of STAT3 ^43^, yet it induces some genes, such as *Tbx21* encoding T-Bet and *Ifng*, via STAT1, with STAT3 opposing their induction ^100^. Differential STAT utilization thus represents a mechanism for fine-tuning the signaling induced by a single cytokine. In addition, IL-2 and IL-21 differentially regulate Th9, Tfh, and Th17 differentiation, providing an example of fine-tuning based on which cytokine is dominant in a given context. Another mechanism of fine-tuning can occur at the level of STAT tetramerization. For example, IL-2 activates STAT5A and STAT5B to form dimers (homodimers and potentially heterodimers) as well as tetramers (potentially both homotetramers and a range of heterotetramers) ^101^. Tetramerization occurs via dimerization of STAT dimers, mediated by an N-terminal region known as the N-domain. Some genes require STAT5 tetramers for their normal expression, whereas STAT5 dimers are sufficient for viability, red cell production, and normal thymic development. Interestingly, STAT5 tetramer-deficient mice have diminished numbers of CD8 and NK cells, defective proliferation, including in response to infection with LCMV, and diminished (albeit not absent) Treg cell function ^101^.

In addition to these physiological mechanisms of fine-tuning signals, IL-2 partial agonists have now been generated. The IL-2 superkine ^68^, which exhibits enhanced binding to IL-2Rβ, was used as a background for selecting mutants that exhibited diminished interaction with γ _c_^69^. In contrast to the super-IL-2 full agonist, such molecules could either abrogate or attenuate IL-2 signaling based on the level of recruitment of γ _c_, thereby altering the E _max_ and signaling threshold. Because these molecules have enhanced binding to IL-2Rβ, they outcompete endogenous IL-2 as well as IL-15 (which also shares IL-2Rβ) and confer a new level of signaling. One such molecule, denoted as H9-RETR, has four amino acid mutations and disrupts the γ _c_ binding interface completely. Not only can this molecule inhibit cytokine-induced STAT5 phosphorylation on T and NK cells but it also inhibits IL-2 or IL-15-induced cytolytic activity of NK cells *in vitro*, prolongs survival in a mouse model of graft-versus-host disease, and inhibits the proliferation of cells from patients with the chronic/smoldering form of human T-cell lymphotropic virus-I (HTLV-I)-induced adult T cell leukemia ^69^. The approach for generating these partial agonists may be able to provide a range of interesting new IL-2 variants and should be broadly applicable to other cytokines as well.

## Concluding remarks

The γ _c_ family of cytokines collectively serve critical roles in the immune system, controlling lymphocyte development, growth, differentiation, and survival. In this review, we have focused primarily on IL-2 and IL-21, clarifying ways in which they regulate the immune response physiologically as well as how they can be utilized and manipulated to modulate the immune system in disease settings. Novel approaches, including the generation of new variants of IL-2 such as an IL-2 superkine or IL-2 partial agonists or the “stabilization” of IL-2 with anti-IL-2 antibodies with effects on binding specificity, show promise for modulating the actions of IL-2 and potentially IL-15 to therapeutic benefit.
